# Association of Air Pollution and Weather Factors with Traffic Injury Severity: A Study in Taiwan

**DOI:** 10.3390/ijerph19127442

**Published:** 2022-06-17

**Authors:** Ta-Chien Chan, Chih-Wei Pai, Chia-Chieh Wu, Jason C. Hsu, Ray-Jade Chen, Wen-Ta Chiu, Carlos Lam

**Affiliations:** 1 Research Center for Humanities and Social Sciences, Academia Sinica, Taipei 11529, Taiwan; dachianpig@gmail.com; 2 Institute of Public Health, School of Medicine, National Yang Ming Chiao Tung University, Taipei 11221, Taiwan; 3 Graduate Institute of Injury Prevention and Control, College of Public Health, Taipei Medical University, Taipei 11031, Taiwan; cpai@tmu.edu.tw (C.-W.P.); wtchiu.tmu@gmail.com (W.-T.C.); 4 Emergency Department, Wan Fang Hospital, Taipei Medical University, Taipei 11696, Taiwan; setfreej@gmail.com; 5 Department of Emergency, School of Medicine, College of Medicine, Taipei Medical University, Taipei 11031, Taiwan; 6 International PhD Program in Biotech and Healthcare Management, College of Management, Taipei Medical University, Taipei 10675, Taiwan; jasonhsu@tmu.edu.tw; 7 Clinical Data Center, Office of Data Science, Taipei Medical University, Taipei 10675, Taiwan; 8 Research Center of Data Science on Healthcare Industry, College of Management, Taipei Medical University, Taipei 10675, Taiwan; 9 Division of General Surgery, Department of Surgery, Taipei Medical University Hospital, Taipei 11031, Taiwan; rayjchen@tmu.edu.tw; 10 Professional Master Program in Artificial Intelligence in Medicine, College of Medicine, Taipei Medical University, Taipei 10675, Taiwan; 11 AHMC Health System, Alhambra, CA 91801, USA

**Keywords:** road traffic injury, injury severity, fine particulate matter, air quality index, active commuters

## Abstract

Exposure to air pollutants may elevate the injury severity scores (ISSs) for road traffic injuries (RTIs). This multicenter cross-sectional study aimed to investigate the associations between air pollution, weather conditions, and RTI severity. This retrospective study was performed in Taiwan in 2018. The location of each road traffic accident (RTA) was used to determine the nearest air quality monitoring and weather station, and the time of each RTA was matched to the corresponding hourly air pollutant concentration and weather factors. Five multiple logistic regression models were used to compute the risk of sustaining severe injury (ISS ≥ 9). Of the 14,973 patients with RTIs, 2853 sustained severe injury. Moderate or unhealthy air quality index, higher exposure to particulate matter ≤2.5 μm in diameter, bicyclists or pedestrians, greater road width, nighttime, and higher temperature and relative humidity were significant risk factors for severe injury. Exposure to nitrogen oxide and ozone did not increase the risk. Auto occupants and scene-to-hospital time were the protective factors. Sensitivity analyses showed consistent results between air pollutants and the risk of severe injury. Poor air quality and hot and humid weather conditions were associated with severe RTIs. Active commuters were at higher risk of sustaining severe RTI.

## 1. Introduction

Air pollutants, such as particulate matter ≤2.5 μm in diameter (PM_2.5_), are harmful to human health, especially the respiratory, cardiovascular, and nervous systems [1]. An increasing number of studies have elucidated the detrimental effects of air pollutants on various aspects of human health. Air pollution exposure is significantly associated with impairment of neurobehavioral performance among Chinese children [2] and adversely affects the cognitive performance of high school students in Israel [3] and Iran [4]. According to studies conducted in the United Kingdom (UK) and the United States (US), acute air pollution exposure may be associated with increased aggressive behaviors [5,6]. Acute exposure causes elevated levels of serotonin [7] and stress hormones [8], including cortisol, cortisone, and epinephrine, resulting in impulsive and aggressive behavior. In addition, direct stimulation of the eyes and upper respiratory tract by air pollutants may cause impatience and bad mood [9].

Road traffic injuries (RTIs) are the eighth leading cause of death worldwide and the number one cause of death among people aged 5–29 years [10]. Environmental conditions are risk factors contributing to road traffic accidents (RTAs) [11,12,13]. Recent studies have reported the associations between RTIs and various weather conditions such as temperature, precipitation, and humidity [9,14,15,16,17]. However, only a few studies have examined the association between air pollution and RTIs. Previous studies have suggested that air pollution increases the risk of death due to unintentional injury, but findings regarding the effect of air pollution on injury severity have varied [18,19]. However, these studies included patients with all-cause injuries rather than focusing on patients with RTIs. In a study conducted in Pakistan, air pollution was determined to cause RTAs [9]. In the UK, higher PM_2.5_ concentrations were associated with higher numbers of car crashes owing to their negative effect on driving performance [20]. In this study, nitrogen dioxide (NO_2_), sulfur dioxide (SO_2_), and air quality index (AQI), but not ozone (O_3_), were also significantly associated with the number of car crashes. However, a previous study conducted in Iran found that O_3_, particulate matter ≤10 μm in diameter (PM_10_), NO_2_, and carbon monoxide (CO) concentrations were inversely associated with mortality after adjusting the confounders [21]. Another study conducted in Iran indicated that the concentration of PM_2.5_ was significantly associated with the number of traffic accidents. However, no significant correlation was observed between NO_2_ and the number of traffic accidents [22]. The association between air pollutants and RTI or mortality was examined in the above studies, which reported inconsistent results.

In Taiwan, ambient air pollution primarily originates from three sources: long-range transport of air pollutants from other countries, emissions from local factories and construction, and traffic-related air pollution. According to a study conducted in 2019, on-road vehicles were the primary source of PM_2.5_ and nitrogen oxide (NO_x_) emissions. The annual average PM_10_ and PM_2.5_ concentrations were 35.7 μg/m^3^ and 17.3 μg/m^3^, respectively. For O_3_ and NO_2_, their annual average concentrations were 31.4 parts per billion (ppb) and 11.5 ppb, respectively [23]. As of 2021, Taiwan has more than 14,000,000 motorcycles, which is one of the major sources of ambient air pollution [24,25]. For the Taiwanese, these motorcycles, mostly light motorcycles with cylinder capacities of less than 250 cc, are used for commuting and supporting daily activities. The high demand for motorcycles is mainly due to their affordability, usage convenience, and other factors, including increased urban traffic congestion, insufficient public transportation systems, and socioeconomic conditions [26,27]. The widespread use of motorcycles has caused road safety issues. More than 474,000 RTIs and 2990 deaths were recorded in 2021; more than 75% of the casualties were motorcyclists. The RTI rate increased by 109.7% from 2008 to 2021 [28].

Driving performance is a key component of road safety, and a driver’s cognitive and behavioral capacities affect the driving performance. Air pollution negatively affects the cognitive and behavioral capacities, thus causing impulsivity, irritability, and aggression [29,30]. Air pollution also causes haze that diminishes the visibility of roads [31]. Therefore, the association between air pollution, RTAs, and related injuries should be explored to ensure road traffic safety [32]. As studies exploring these associations are limited, increased air pollution concentrations might cause more severe traffic injuries. The primary objective of this multicenter study was to investigate the associations of air pollution, including six traffic-related air pollutants, and the composite index (AQI) and weather factors with RTI severity.

By exploring the association of meteorological factors and air pollution with RTIs, our study provides useful information for the healthcare authorities in planning effective interventions to reduce deaths and injuries caused by RTAs. In addition, this exploration may open a new pathway for trauma research whose results can be applied in a clinical setting in order to mitigate the severity of RTIs. For the transport sector, it is important to maintain a convenient public transport system that can decrease the need for private transport, improve road congestion, alleviate environmental pollution, and decrease the injury severity after RTAs. Combining the transport and environmental sectors’ efforts in urban and environmental planning will lead to the implementation of the concept of green transportation, including electric vehicles, ride-sharing, and green space. Urban greenery is also an effective strategy for mitigating air pollution [33]. These interventions will be associated to reducing the air pollution and the severity of RTIs. This study echoes the United Nations’ Sustainable Development Goal 2030 of reducing global deaths and injuries from RTAs (Target 3.6) and substantially reducing deaths and illnesses caused by air pollution and contamination (Target 3.9) [34].

## 2. Materials and Methods

### 2.1. Study Design

**Figure 1:**
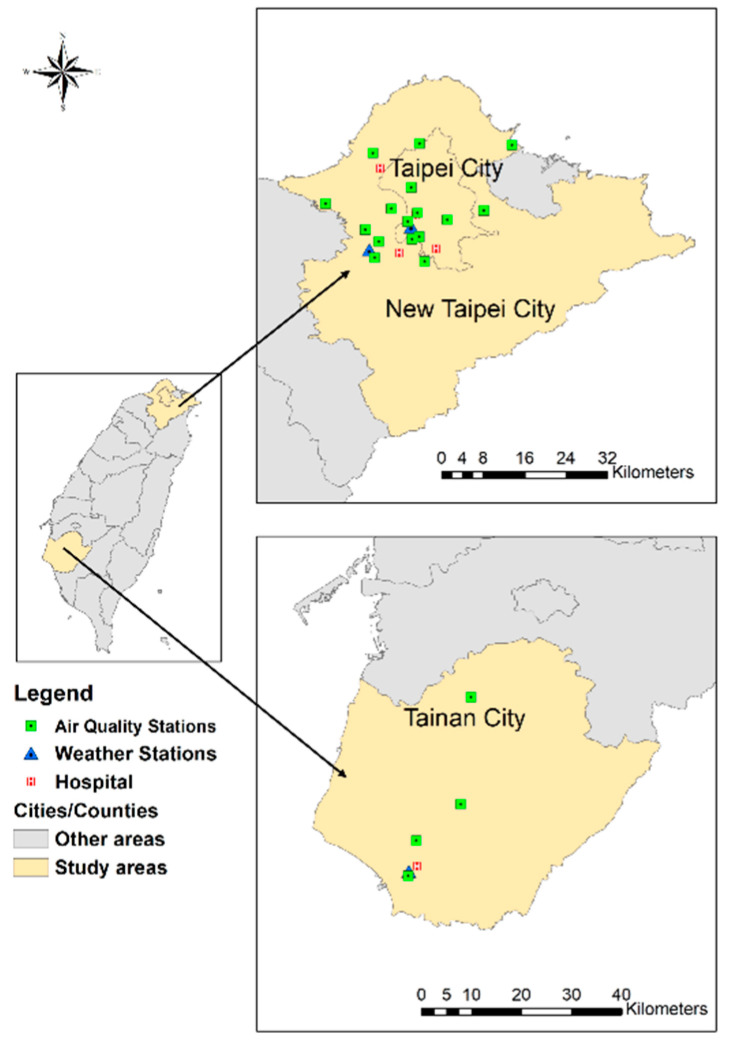
Geographical locations of participating hospitals and monitoring stations.

A multicenter cross-sectional study was performed involving patients from five trauma centers in Taiwan: Shuang Ho Hospital, Wan Fang Hospital, the Taipei and Tamsui branches of Mackay Memorial Hospital, and the National Cheng Kung University Hospital. The locations of these hospitals are shown in Figure 1. Mackay Memorial Hospital (Taipei branch) and Wan Fang Hospital are located in the central and southern regions of Taipei City, with 210,447 and 256,942 residents, respectively. Mackay Memorial Hospital (Tamsui branch) and Shuang Ho Hospital are located in the northwest and southwest regions of New Taipei City, with 184,660 and 401,086 residents, respectively. National Cheng Kung University Hospital is located in the northern region of Tainan City, which has 126,579 residents. All five hospitals are university-affiliated teaching hospitals and qualified as advanced emergency responsibility hospitals in Taiwan, equivalent to trauma centers in the US that provide comprehensive care for trauma patients. This study was conducted in accordance with the Strengthening the Reporting of Observational Studies in Epidemiology guidelines (Table S1). This study was approved by the institutional review boards of the participating hospitals (16MMHIS168e, N201510012, and A-ER-105-401).

**Figure 2:**
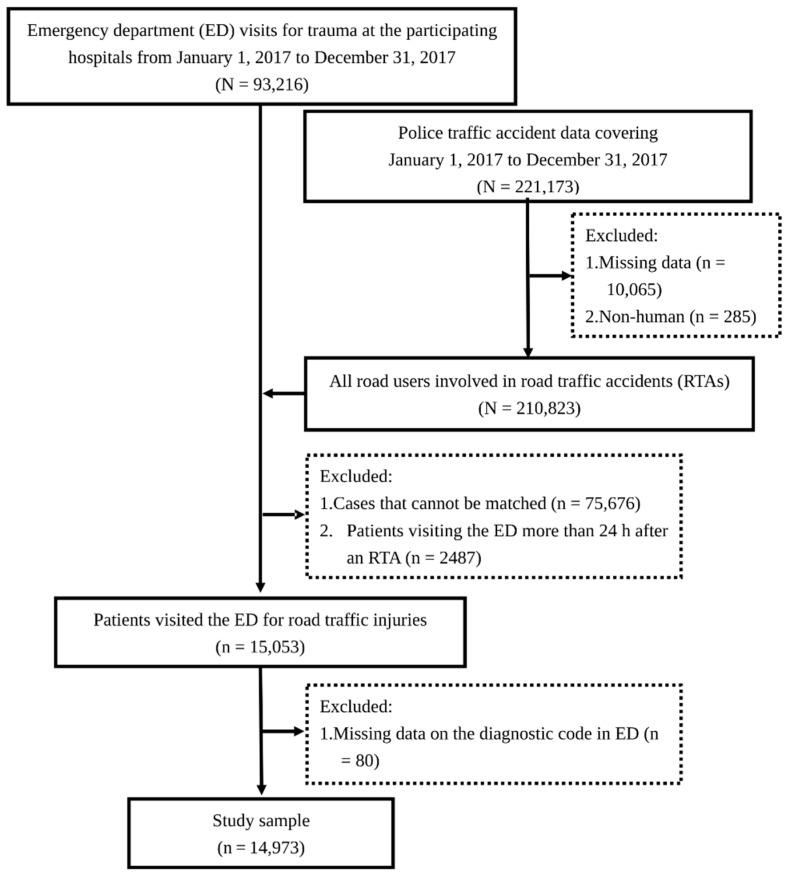
Flow chart showing the sample selection process. ED, emergency department; RTA, road traffic accident.

Data were retrospectively collected from the electronic medical records of patients with RTIs treated at the aforementioned trauma centers between 1 January 2017 and 31 December 2017. The patients’ national identification numbers were used to connect the collected data to the police traffic accident dataset (PTAD) in order to identify patients whose RTIs were treated in the emergency department (ED) and obtain information regarding the RTAs in which they were involved. The identification numbers were deleted upon connection. To further ensure that RTAs and ED visits were causally related, we only included patients admitted in the ED within 24 h after an RTA. For each patient who visited the ED several times after sustaining an RTI, only the records of the initial visit were included. The patient selection process is illustrated in Figure 2.

The PTAD, which included coded information of the crashes and the involved personnel, was compiled from records created by on-scene police officers. The PTAD contained information on the road user types and other conditions, including the time of the RTA, the involved driver, and the involved vehicle, which may have been related to each RTA described therein. The roadway data included information on the location of each RTA, while the accident-related data included information on contributing circumstances, driver/vehicle actions, and collision types.

### 2.2. Measurements

The following demographic and environmental factors were retrieved from the PTAD: sex, age (divided into four groups: <24, 25–44, 45–64, and ≥65 years), day of the crash (weekday or weekend), time of the crash (daytime (06:00 to 17:59), evening (18:00 to 23:59), or nighttime (00:00 to 05:59)), rush hour (07:00 to 09:00, 17:00 to 19:00) or non-rush hours, and road user type (auto occupants, motorcyclists, bicyclists, or pedestrians). Data on the injury severity score (ISS), ED arrival time, and Glasgow Coma Scale (GCS) score (which measures the level of consciousness) were retrieved from the hospital data. The scene-to-hospital arrival time (min) was calculated to determine the delay between the accident time and time of arrival at the hospital.

The air pollutants examined in this study included hourly concentrations of PM_2.5_, PM_10_, O_3_, NO_2_, and NO_x_.

The hourly AQI was also assessed, which was determined based on the maximum subindex value among the concentrations of the following air pollutants: PM_2.5_, PM_10_, O_3_, SO_2_, CO, and NO_2_ [35]. The AQI ranges from 0 to 500, with a higher score indicating poorer air quality. The AQI values were divided into three groups according to the Environmental Protection Administration regulations: good (0–50), moderate (51–100), and unhealthy (>100) [36].

In terms of the location of the crash scene, only the address was included in its corresponding record in PTAD. Geographic information system platforms, including Google Maps application programming interfaces [37] and Taiwan Geospatial One Stop [38], were used to convert each address into geographical coordinates. The air quality data in each crash scene from the nearest air monitoring station, which can be downloaded openly from the Taiwan Environmental Protection Administration [39], were collected. Data were collected from 18 stations, comprising 15 ambient monitoring stations and three traffic monitoring stations. The road width at each crash scene, computed from a commercial digital map using ArcGIS 10.2 (ESRI, Redlands, CA, USA), was used as a surrogate measure of local traffic flow volume.

The data regarding weather conditions at the time of RTA, such as hourly mean temperature (°C), hourly mean relative humidity (%), and 24-h accumulated precipitation (mm), were obtained from the Taiwan Central Weather Bureau [40]. The 24-h accumulated precipitation amounts were divided into two levels based on the Central Weather Bureau’s definition of heavy rain: <80 mm and ≥80 mm.

The hours of each RTA were matched to the corresponding hourly air pollutant concentration, AQI, temperature, and relative humidity. The cumulative precipitation within 24 h preceding each RTA event was recorded.

### 2.3. Outcome

The ISS was used as an outcome measure. Although the patients’ ISSs are not mandatorily recorded in EDs in Taiwan, the International Classification of Diseases, Tenth Revision, Clinical Modification (ICD-10-CM) codes are universally used to record the diagnoses of patients when they are discharged from the ED. A previously validated package (ICDPIC-R) for R statistical software [41] was used to translate the ICD-10-CM codes from the hospital data into ISSs. Because the majority of RTIs in Taiwan are not life-threatening injuries [42] and because morbidity (rather than mortality) was the primary outcome of interest in our study, the patients’ ISSs were divided into <9 points and ≥9 points; an ISS of ≥9 indicated severe injury requiring hospitalization or intensive care [43,44,45].

### 2.4. Statistical Analysis

A univariate analysis was conducted to evaluate the association between each independent variable and the outcome measure. Pearson’s Chi-square test, Cochran–Armitage trend test, and Wilcoxon rank-sum test were used to analyze the categorical, ordered categorical, and continuous variables, respectively. Because the dependent variable was binary (ISS < 9 vs. ≥9 points), a multiple logistic regression analysis was performed using the patient’s sex, age, and the independent variables with a *p* value of <0.2 in the univariate analysis [46]. However, as previous studies have identified GCS as a surrogate variable for injury severity [47,48], GCS was removed from the multiple regression model.

The binary logistic regression model has been commonly estimated in the trauma or traffic injury literature [45,49,50] to identify the determinant of the outcome variable of interest, which was dichotomous. In this study, the outcome response of interest (severe injuries (ISS ≥ 9) vs. non-severe injuries (ISS < 9)) was binary. In contrast to the ordinary least-squares regression model, the dependent variable in the binary logistic regression model is not limited by the assumptions of a continuous or normal distribution. Binary logistic regression is a regression model used to estimate the association between a set of independent variables, whether categorical or continuous, and binary outcomes.

In the binary logistic regression model, the equation is formulated as follows:

(1) $$g\left(x\right)=\beta_{0}+\beta_{1}x_{1}+\beta_{2}x_{2}+\ldots +\beta_{p}x_{p}$$

where *x_j_* is the value of the *j*th independent variable, *β_j_* is the corresponding coefficient for *j* = 1, 2, 3,…, *p*, and *p* is the number of independent variables.

The conditional probability of a positive outcome given the independent variable is as follows:

(2) $$\pi \left(x\right)=\frac{\exp(g\left(x\right))}{1+\exp(g\left(x\right))}$$

The maximum likelihood method was used to estimate the parameters of the logistic regression model by constructing the likelihood function:

(3) $$l\left(\beta \right)=\prod_{i=1}^{n}\pi {\left(x_{i}\right)}^{y_{i}}{\left(1-\pi \left(x_{i}\right)\right)}^{1-y_{i}}$$

where *y_i_* denotes the *i*th observed outcome with a value of either 0 or 1 and *i* = 1, 2, 3,…, *n*, where *n* is the number of observations. The best regression estimate of *β* was determined by maximizing the log-likelihood expression:

(4) $$LL\left(\beta \right)=\ln(l\left(\beta \right))=\sum_{i=1}^{n}\left\{y_{i}\ln(\pi \left(x_{i}\right))+\left(1-y_{i}\right)\ln(1-\pi \left(x_{i}\right))\right\}$$

The exponentiated coefficient exp(*β_j_*), odds ratio (OR), is usually interpreted for a logistic regression model to reveal the effect of attributes on the likelihood of severe injuries:

(5) $$\mathrm{OR}=\exp(\beta_{j})$$

with a 95% confidence interval (CI) of (exp(*β_j_* − 1.96*sβ_j_*), exp(*β_j_* + 1.96*sβ_j_*)), where *sβ* is the standard error of coefficient *β*. An OR of >1 indicated a positive association between the interest attribute and severe injuries, whereas an OR of <1 indicated a negative association between the interest attribute and severe injuries. An OR of 1 indicated that no association was found between the interest attributes and severe injuries.

To assess the model’s fit to the data and determine the best-fit model in multiple models, the Akaike information criterion (AIC) was defined as follows:

(6) AIC=−2ln(L)+2k

where *L* is the log-likelihood estimate, and *k* is the number of independent variables. A lower AIC score indicated a better fit for the model.

As the AQI was determined according to the maximum subindex values of the PM_2.5_, PM_10_, O_3_, SO_2_, CO, and NO_2_ concentrations, a separate model was required to avoid collinearity problems in the multivariable model. In addition, considering that strong correlations between PM_2.5_ and PM_10_ (*r* = 0.832, *p* < 0.001) and between NO_2_ and NO_x_ (*r* = 0.968, *p* < 0.001) were identified in the multiple air pollutant model, these air pollutants were entered separately in five multivariable models (Model 1: AQI; Model 2: PM_2.5_, NO_x_, and O_3_; Model 3: PM_2.5_, NO_2_, and O_3_; Model 4: PM_10_, NO_x_, and O_3_; and Model 5: PM_10_, NO_2_, and O_3_). The variance inflation factor (VIF) was used for identifying collinearity; a VIF value of <5 was considered acceptable during variable selection. A sensitivity analysis was further conducted by removing the auto-occupant patients.

The ORs and 95% CIs were calculated to estimate the risk of sustaining severe injuries. A two-sided *p* value of <0.05 was considered significant. Statistical analyses were restricted to patients with no missing values for each specific variable and were performed using the SAS software (version 9.4; SAS Institute, Cary, NC, USA).

## 3. Results

A total of 14,973 patients with RTIs treated at the EDs of the five participating hospitals were identified; of them, 2853 patients (19.1%) sustained severe injury (ISS ≥ 9 points). The patient’s mean age was 37 years, and 54.6% of them were men.

The results of univariate analysis are presented in Table 1. Significant differences were observed in age, GCS, scene-to-hospital arrival time, time of crash, type of road user, road width, 24-h accumulated precipitation, temperature, relative humidity, AQI, and PM_2.5_, PM_10_, NO_2_, and NO_x_ concentrations.

Results of the multiple air pollutant model are listed in Table S2. All multivariable models consistently demonstrated that air pollution and other weather and environmental factors were significantly associated with injury severity among patients. The AQI and best-fit multiple air pollution models are presented in Table 2.

In the AQI model, compared with patients aged <24 years, those aged 45–64 years or >64 years were more likely to have an ISS of ≥9 (OR: 1.398, 95% CI: 1.247–1.567 and OR: 1.767, 95% CI: 1.517–2.058, respectively). Involvement in a nighttime crash was a risk factor for obtaining an ISS of ≥9 (OR: 1.491, 95% CI: 1.254–1.774). The risk of obtaining an ISS of ≥9 was significantly higher among patients involved in RTAs on wider roads (OR: 1.014, 95% CI: 1.009–1.019). Compared with motorcyclists, bicyclists and pedestrians were more likely to have an ISS of ≥9 (OR: 1.457, 95% CI: 1.183–1.795 and OR: 1.379, 95% CI: 1.180–1.612, respectively), whereas auto occupants were less likely to sustain severe injuries (OR: 0.185, 95% CI: 0.133–0.256). With regard to weather conditions, the risk of obtaining an ISS of ≥9 increased significantly with every interquartile range (IQR) increase in temperature (OR: 1.238, 95% CI: 1.146–1.338) and relative humidity (OR: 1.195, 95% CI: 1.117–1.278). Moderate and unhealthy AQIs were risk factors for an ISS ≥9 (OR: 1.142, 95% CI: 1.040–1.253 and OR: 1.713, 95% CI: 1.486–1.975, respectively). The risk of obtaining an ISS of ≥9 was lower among patients with shorter scene-to-hospital arrival times (OR: 0.999, 95% CI: 0.998–0.999).

In the multiple air pollutant model (Table 2), the risk of obtaining an ISS of ≥9 was higher among patients aged 45–64 or ≥65 years (OR: 1.382, 95% CI: 1.228–1.555 and OR: 1.757, 95% CI: 1.502–2.055, respectively) than among patients aged <24 years. Involvement in nighttime crashes was a risk factor for sustaining severe injury (OR: 1.400, 95% CI: 1.166–1.680). Compared with motorcyclists, bicyclists and pedestrians were more likely to have an ISS of ≥9 (OR: 1.429, 95% CI: 1.154–1.770 and OR: 1.412, 95% CI: 1.202–1.660, respectively), whereas auto occupants were at a lower risk of obtaining an ISS of ≥9 (OR: 0.180, 95% CI: 0.128–0.253). Each IQR increment in temperature and relative humidity was associated with a significant increase in the risk of obtaining an ISS of ≥9 (OR: 1.165, 95% CI: 1.077–1.262 and OR: 1.136, 95% CI: 1.054–1.224, respectively). With regard to air pollutants, each IQR increase in PM_2.5_ concentration was also associated with a significant increase in the risk of sustaining severe injury (OR: 1.279, 95% CI: 1.209–1.353). NO_x_ and O_3_ concentrations were negatively associated with an ISS of ≥9. IQR increments in NO_x_ or O_3_ concentrations were not associated with increases in the risk of obtaining an ISS of ≥9 (OR: 0.743, 95% CI: 0.696–0.794 and OR: 0.838, 95% CI: 0.774–0.906, respectively). The risk of obtaining an ISS of ≥9 was lower among patients with shorter scene-to-hospital arrival times (OR: 0.998, 95% CI: 0.998–0.999).

Finally, the sensitivity analysis results, including five models (Table S3), revealed that the association between air pollutants and the risk of obtaining an ISS of ≥9 was consistent with the findings of the multivariate analysis.

## 4. Discussion

To the best of our knowledge, no large epidemiological studies have elucidated the associations between air pollution, other weather and environmental conditions, and RTI severity. A high resolution of the temporal and spatial information collected in this study is crucial for clarifying such associations. The time between environmental exposure and the occurrence of RTA is often short, and the incident time is correlated with different risk factors. The precise location of a crash can be used to investigate the road features, compute the transportation time from the scene to the hospital, and identify the nearest weather or air quality monitoring station. Furthermore, previous studies have mostly focused on investigating RTAs rather than on the RTI severity. Analyzing the injury burdens associated with different ISSs is useful for formulating appropriate public health policies.

Active commuters (pedestrians and cyclists) inhale higher doses of air pollutants compared with commuters who use motorized transport [51]. A previous study in China revealed that PM_10_ and PM_2.5_ concentrations are correlated with RTA rates [32]. In the UK, one study identified a 0.3–0.6% increase in the number of vehicles involved in RTAs per day for each 1 μg/m^3^ increment in PM_2.5_ concentration [20]. The present study revealed that unhealthy AQIs were associated with an increased risk of sustaining severe RTIs. In addition, the patients involved in RTAs that occurred in periods with higher hourly PM_2.5_ concentrations were more likely to sustain severe RTIs. To our knowledge, this study was the first to address the association between air pollutant exposure and RTI severity. For active commuters, a higher inhalation rate and longer commuting time increase the amount of air pollutant exposure. Among all traffic-related air pollutants, exposure to PM_2.5_, the primary pollutant emitted from vehicles, can lead to acute and chronic cardiovascular injuries [52]. In addition, outdoor air pollution exposure might affect the central nervous system (CNS), impair cognitive function, and increase the incidence of stroke and other neuropathological problems in humans [53]. The possible biological mechanisms that trigger RTIs include acute cardiovascular or CNS disorders. Further studies are required to explore these mechanisms.

An inverse association was observed between NO_x_ and O_3_ concentrations and the risk of sustaining severe injury. A possible reason for this association is the lagged effect of NO_2_ (NO_x_) and O_3_, as they are secondary and photochemical oxidants [54]. In this study, the time of each crash was linked to the corresponding hourly concentration of air pollutants; therefore, only primary pollutants, such as PM_2.5_, PM_10_, or those with the maximum sub-index values (reflected using the AQI), were clearly associated with RTI severity. The existing strategies to reduce air pollution in urban environments have mainly focused on the reforming sustainable transport systems to reduce the health risks of air pollution (https://www.who.int/publications/i/item/WHO-HEP-ECH-AQH-2021.6 accessed on 3 May 2022). Urban planning of greenspaces might also help reduce the air pollution levels. Green space plays a role in improving air quality through particle deposition, dispersion, and modification [55]. Another study conducted in China showed that the overall abundance of green spaces in urban areas can significantly reduce the PM_10_ concentrations [56]. Green space might play a role in mitigating the air pollutant levels and indirectly reduce the incidence of RTA.

A previous study using the National Health Insurance database and National Traffic Crash Dataset in Taiwan from 2003 to 2012 revealed that bicyclists were at a higher risk of sustaining severe injury than motorcyclists (OR: 1.11, 95% CI: 1.08–1.14) [57]. The present study also discovered that bicyclists were at a higher risk of sustaining severe injury (OR: 1.5) compared with motorcyclists. The main reason for this is that the helmet utilization rate among cyclists is low, whereas that among motorcyclists is high, particularly because helmets have been required by law for motorcyclists since 1997.

Weather conditions may be associated with the incidence or severity of RTIs. Under hot and humid conditions, crashes may be attributed to the changes in the behavior of drivers or pedestrians. On hot days, limited skill or power performance may cause tiredness and impaired effectiveness, resulting in a higher risk of accidents [58]. However, under extreme weather conditions, such as heatwaves, people may adopt protective behaviors, resulting in fewer accidents [58]. One study in Hong Kong utilized fine-resolution weather data (1-h interval) to determine the correlation between weather factors and traffic crashes [59]. Results indicated that the maximum relative risks of high temperature, humid weather, and precipitation were 1.11, 1.14, and 1.34, respectively. Another study conducted in Nebraska, USA, indicated that rain and warmer air temperatures were associated with more severe crash injuries in single-vehicle truck crashes [60]. However, humidity is inversely associated with injury severity. Another study on the effects of weather conditions on pedestrian injury severity reported that high temperatures and rainfall were associated with fatal and severe injuries [61]. In our study, high temperature and high relative humidity were associated with severe injuries. The cumulative 24-h rainfall before a crash was insignificant in the final model.

At night, poor visibility may result in a higher risk of RTI and RTA mortality. A 5-year epidemiological study in Ghana revealed that the relative risk of mortality in a nighttime traffic crash was 1.3 times higher than that in a daytime traffic crash [62]. The present study showed that individuals involved in RTAs late at night (0:00 to 5:59) were at a 1.5 times higher risk of sustaining severe injury than those involved in RTAs during the day. Low light levels result in delayed reaction times [63]. A cross-sectional study in Bangladesh indicated that patients who sustained from RTAs that occurred after midnight tended to have higher ISSs [64].

Age is an important risk factor for RTIs, particularly in older adults. A previous review indicated that 23.6% of RTIs are sustained by older adults [65]. In addition, the clinical severity of RTIs and the mortality rate associated with RTAs are relatively high among older adults [66]. These findings are consistent with our results, in which individuals aged ≥65 years were at the highest risk of sustaining severe injury (ISS ≥ 9). Middle-aged adults (45–64 years) had the second-highest risk of sustaining severe injury.

This multicenter approach strengthened the generalizability of our results. The findings of this study were derived from the data of 14,973 patients with RTIs, making our analysis the first with a sufficient sample size to report an association between air pollution and injury severity in patients with RTIs. To assess for collinearity, five models were established for different air pollutants to more effectively demonstrate the association between air pollution and RTI severity. Finally, the consistency of the sensitivity test results among the five models proved the reliability of the study results. Our study provides useful information for the prevention and control of RTIs.

### Limitations

This study has some limitations. First, exposure to air pollution might have triggered the acute exacerbations of underlying diseases and resulted in severe RTIs. However, the underlying diseases of these patients are unknown. Nevertheless, age can be used as a surrogate measure. Second, the lower risk of sustaining severe RTIs among motorcyclists may be attributable to the increased utilization of helmets among the motorcyclists in Taiwan. In this study, information on helmet use among the cyclists was not obtained. Third, this analysis focused on investigating the association between acute pollution exposure and RTI severity. However, if the modes of transport remain similar from day to day, the cumulative effects of air pollution exposure on RTI severity may also be possible. Finally, data on drug use were lacking, and 18% of the data related to alcohol consumption among victims was missing. Therefore, these two factors were not included in our analysis.

## 5. Conclusions

Male sex, older age, and time of crash being nighttime were associated with a higher risk of sustaining severe RTI. Active commuters, including bicyclists and pedestrians, are at higher risk of sustaining severe RTI. Poor air quality measured based on the AQI or PM_2.5_ concentration, and hot and humid weather conditions are all associated with severe RTIs; therefore, real-time environmental monitoring data should be incorporated into the traffic warning systems. This study highlights the positive associations between RTI, air pollution, and weather factors. Further in-depth evaluation of the aforementioned air pollution factors may yield valuable insights for the prevention of severe RTIs.

## Figures and Tables

**Table 1:** Summary of independent variables for patients with ISSs of <9 and ≥9.

Variables	ISS < 9 (*n* = 12,120)	ISS ≥ 9 (*n* = 2853)	*p* Value
	*n* (%)	*n* (%)	
Sex			0.26 ^a^
Male	6591 (89.61)	1585 (19.39)	
Female	5529 (81.34)	1268 (18.66)	
Age (year) median (IQR)	30 (27)	36 (34)	<0.001 ^b^
Age group (year)			<0.001 ^c^
<24	4541 (82.80)	943 (17.20)	
25–44	4037 (83.31)	809 (16.69)	
45–64	2596 (77.86)	738 (22.14)	
>64	946 (72.27)	363 (22.73)	
GCS median (IQR)	15 (0)	15 (0)	<0.001 ^b^
Scene-to-hospital arrival time (min) median (IQR)	35 (42)	31 (18)	<0.001 ^b^
Day of crash			0.42 ^a^
Weekday	8910 (81.10)	2076 (18.90)	
Weekend	3210 (80.51)	777 (19.49)	
Time of crash (time of day)			<0.001 ^a^
Day (06:00–17:59)	8040 (81.03)	1882 (18.97)	
Evening (18:00–23:59)	3364 (81.77)	750 (18.23)	
Night (00:00–05:59)	716 (76.41)	221 (23.59)	
Time of crash (rush hour)			0.93 ^a^
07:00–09:00	1741 (80.83)	413 (19.17)	
17:00–19:00	1647 (80.70)	394 (19.30)	
Nonrush hours	8732 (81.02)	2046 (18.98)	
Type of road user			<0.001 ^a^
Auto occupant	866 (95.37)	42 (4.63)	
Motorcyclist	10,117 (80.95)	2381 (19.05)	
Bicyclist	364 (71.79)	143 (28.21)	
Pedestrian	771 (73.08)	284 (26.92)	
Missing ^d^	2 (0.02)	3 (0.11)	
Road width (m) median (IQR)	10 (10)	11 (11)	<0.001 ^b^
Missing ^d^	9 (0.07)	2 (0.07)	
24-h accumulated precipitation (mm) Median (IQR)	0 (2.5)	0 (2)	0.008 ^b^
Heavy rain			0.64 ^a^
No (<80 mm)	12,006 (80.97)	2821 (19.03)	
Yes (≥80 mm)	112 (79.43)	29 (20.57)	
Missing data ^d^	2 (0.02)	3 (0.11)	
Temperature (℃) median (IQR)	25 (10)	26 (10)	0.006 ^b^
Relative humidity (%) median (IQR)	71 (15)	71 (15)	0.010 ^b^
AQI median (IQR)	51 (38)	53.5 (43)	<0.001 ^b^
AQI level			<0.001 ^c^
Good (0–50)	5698 (82.04)	1247 (17.96)	
Moderate (51–100)	4845 (81.10)	1129 (18.90)	
Unhealthy (>100)	1117 (75.83)	356 (24.17)	
Missing data ^d^	460 (3.80)	121 (4.24)	
PM_2.5_ (μg/m^3^) median (IQR)	16 (15)	16 (17)	0.003 ^b^
Missing data ^d^	653 (5.19)	141 (4.94)	
PM_10_ (μg/m^3^) median (IQR)	33 (26)	35 (28)	<0.001 ^b^
Missing data ^d^	482 (3.98)	106 (3.79)	
NO_2_ (ppb) median (IQR)	15 (14.4)	14 (13.4)	<0.001 ^b^
Missing data ^d^	588 (4.85)	140 (4.91)	
NO_x_ (ppb) median (IQR)	19 (19)	16 (18)	<0.001 ^b^
Missing data ^d^	762 (6.29)	188 (6.59)	
O_3_ (ppb) median (IQR)	29 (25)	29 (25.5)	0.49 ^b^
Missing data ^d^	512 (4.22)	121 (4.24)	

^a^ Pearson’s Chi-square test; ^b^ Wilcoxon rank-sum test; ^c^ Cochran–Armitage trend test; ^d^ Note the number of data points with missing data (percentage of all data points with missing data). AQI, air quality index; GCS, Glasgow Coma Scale; ISS, injury severity score; IQR, interquartile range; NO_2_, nitrogen dioxide; NO_x_, nitrogen oxide; O_3_, ozone; PM_2.5_, particulate matter ≤2.5 μm in diameter; PM_10_, particulate matter ≤10 μm in diameter; ppb, parts per billion.

**Table 2:** Results of multiple regression analysis of risk factors and ISS ≥ 9.

Variables	AQI Model		Best-Fit Multiple Air Pollutant Model	
	OR (95% CI)	*p* Value	OR (95% CI)	*p* Value
Sex				
Female	Reference		Reference	
Male	1.102 (1.010–1.202)	0.028	1.108 (1.013–1.211)	0.025
Age group (year)				
<24	Reference		Reference	
25–44	1.023 (0.920–1.139)	0.67	1.046 (0.937–1.168)	0.42
45–64	1.398 (1.247–1.567)	<0.001	1.382 (1.228–1.555)	<0.001
>64	1.767 (1.517–2.058)	<0.001	1.757 (1.502–2.055)	<0.001
Time of crash				
Day (06:00–17:59)	Reference		Reference	
Evening (18:00–23:59)	0.975 (0.882–1.078)	0.62	1.033 (0.932–1.144)	0.54
Night (00:00–05:59)	1.491 (1.254–1.774)	<0.001	1.400 (1.166–1.680)	<0.001
Scene-to-hospital arrival time (min)	0.999 (0.998–0.999)	<0.001	0.998 (0.998–0.999)	<0.001
Type of road user				
Motorcyclist	Reference		Reference	
Bicyclist	1.457 (1.183–1.795)	0.001	1.429 (1.154–1.770)	0.001
Pedestrian	1.379 (1.180–1.612)	<0.001	1.412 (1.202–1.660)	<0.001
Auto occupant	0.185 (0.133–0.256)	<0.001	0.180 (0.128–0.253)	<0.001
Road width (m)	1.014 (1.009–1.019)	<0.001	1.013 (1.008–1.019)	<0.001
Temperature (°C, per IQR)	1.238 (1.146–1.338)	<0.001	1.165 (1.077–1.262)	<0.001
Relative humidity (%, per IQR)	1.195 (1.117–1.278)	<0.001	1.136 (1.054–1.224)	<0.001
AQI level				
Good (0–50)	Reference			
Moderate (51–100)	1.142 (1.040–1.253)	0.005		
Unhealthy (>100)	1.713 (1.486–1.975)	<0.001		
PM_2.5_ (μg/m^3^, per IQR)			1.279 (1.209–1.353)	<0.001
NO_x_ (ppb, per IQR)			0.743 (0.696–0.794)	<0.001
O_3_ (ppb, per IQR)			0.838 (0.774–0.906)	<0.001

The effect estimate was based on the interquartile range (IQR) increases in temperature, relative humidity, and air pollutant concentration. The IQRs of temperature, relative humidity, PM_2.5_, NO_x_, and O_3_ were 9.7 °C, 15%, 15 μg/m^3^, 19 ppb, and 25 ppb, respectively. AQI, air quality index; CI, confidence interval; ISS, injury severity score; NO_x_, nitrogen oxide; O_3_, ozone; OR, odds ratio; PM_2.5_, particulate matter ≤2.5 μm in diameter; ppb, parts per billion.

## Data Availability

The data that support the findings of this study are available from the corresponding author upon reasonable request.

## Supplementary Materials

The following supporting information can be downloaded at: https://www.mdpi.com/article/10.3390/ijerph19127442/s1, Table S1: STROBE Statement: checklist of items that should be included in reports of cross-sectional studies; Table S2: Results of all multiple air pollutant models; Table S3: Results of sensitivity analysis.
