# Supplementary material for: Association of Air Pollution and Weather Factors with Traffic Injury Severity: A Study in Taiwan
Source: Int J Environ Res Public Health. 2022 Jun 17;19(12):7442. doi: 10.3390/ijerph19127442 (PMC9223547; doi:10.3390/ijerph19127442)
Supplement: Supplementary file 1 [file ijerph-19-07442-s001.zip › ijerph-1743762-supplementary.pdf]

**Table S1.** STROBE Statement: checklist of items that should be included in reports of cross-sectional studies.

| | Item No | Recommendation | Page No |
|---|---|---|---|
| **Title and abstract** | 1 | (*a*) Indicate the study's design with a commonly used term in the title or the abstract | 1 |
| | | (*b*) Provide in the abstract an informative and balanced summary of what was done and what was found | 1 |
| **Introduction** | | | |
| Background/rationale | 2 | Explain the scientific background and rationale for the investigation being reported | 1–3 |
| Objectives | 3 | State specific objectives, including any prespecified hypotheses | 2, 3 |
| **Methods** | | | |
| Study design | 4 | Present key elements of study design early in the paper | 3–5 |
| Setting | 5 | Describe the setting, locations, and relevant dates, including periods of recruitment, exposure, follow-up, and data collection | 3–5 |
| Participants | 6 | (*a*) Give the eligibility criteria, and the sources and methods of selection of participants | 3, 4 |
| Variables | 7 | Clearly define all outcomes, exposures, predictors, potential confounders, and effect modifiers. Give diagnostic criteria, if applicable | 5, 6 |
| Data sources/ measurement | 8* | For each variable of interest, give sources of data and details of methods of assessment (measurement). Describe comparability of assessment methods if there is more than one group | 5, 6 |
| Bias | 9 | Describe any efforts to address potential sources of bias | 3, 5, 6 |
| Study size | 10 | Explain how the study size was arrived at | 3, Figure 2 |
| Quantitative variables | 11 | Explain how quantitative variables were handled in the analyses. If applicable, describe which groupings were chosen and why | 5, 6 |
| Statistical methods | 12 | (*a*) Describe all statistical methods, including those used to control for confounding | 6 |
| | | (*b*) Describe any methods used to examine subgroups and interactions | Not applicable |
| | | (*c*) Explain how missing data were addressed | 6 |
| | | (*d*) If applicable, describe analytical methods taking account of sampling strategy | Not applicable |
| | | (*e*) Describe any sensitivity analyses | 6 |
| **Results** | | | |
| Participants | 13* | (a) Report numbers of individuals at each stage of study—e.g., numbers potentially eligible, examined for eligibility, confirmed | 7 |

| | | | |
|---|---|---|---|
| | | eligible, included in the study, completing follow-up, and analysed | |
| | | (b) Give reasons for non-participation at each stage | Figure 2 |
| | | (c) Consider use of a flow diagram | Figure 2 |
| Descriptive data | 14* | (a) Give characteristics of study participants (e.g., demographic, clinical, social) and information on exposures and potential confounders | Table 1 |
| | | (b) Indicate number of participants with missing data for each variable of interest | Table 1 |
| Outcome data | 15* | Report numbers of outcome events or summary measures | 7 |
| Main results | 16 | (*a*) Give unadjusted estimates and, if applicable, confounder-adjusted estimates and their precision (e.g., 95% confidence interval). Make clear which confounders were adjusted for and why they were included | 7–10 |
| | | (*b*) Report category boundaries when continuous variables were categorized | Table 1, Table 2, Table S2 |
| | | (*c*) If relevant, consider translating estimates of relative risk into absolute risk for a meaningful time period | Not applicable |
| Other analyses | 17 | Report other analyses done—e.g., analyses of subgroups and interactions, and sensitivity analyses | 10, Table S3 |
| **Discussion** | | | |
| Key results | 18 | Summarise key results with reference to study objectives | 10–11 |
| Limitations | 19 | Discuss limitations of the study, taking into account sources of potential bias or imprecision. Discuss both direction and magnitude of any potential bias | 11–12 |
| Interpretation | 20 | Give a cautious overall interpretation of results considering objectives, limitations, multiplicity of analyses, results from similar studies, and other relevant evidence | 10–11 |
| Generalisability | 21 | Discuss the generalisability (external validity) of the study results | 11 |
| **Other information** | | | |
| Funding | 22 | Give the source of funding and the role of the funders for the present study and, if applicable, for the original study on which the present article is based | 12 |

**Source:** The Strengthening the Reporting of Observational Studies in Epidemiology (STROBE) Statement: guidelines for reporting observational studies. https://www.equator-network.org/reporting-guidelines/strobe/.

**Table S2.** Results of all multiple air pollutant models.

| Variables | Model 1 (AQI) | | | Model 2 ($PM_{2.5}$, $NO_X$, $O_3$) | | | Model 3 ($PM_{2.5}$, $NO_2$, $O_3$) | | | Model 4 ($PM_{10}$, $NO_X$, $O_3$) | | | Model 5 ($PM_{10}$, $NO_2$, $O_3$) | | |
|---|---|---|---|---|---|---|---|---|---|---|---|---|---|---|---|
| | **OR** | **95% CI** | ***p*** | **OR** | **95% CI** | ***p*** | **OR** | **95% CI** | ***p*** | **OR** | **95% CI** | ***p*** | **OR** | **95% CI** | ***p*** |
| **AQI level** *(Reference: Good (0–50))* | | | | | | | | | | | | | | | |
| Moderate (51–100) | 1.142 | (1.040–1.253) | 0.005 | | | | | | | | | | | | |
| Unhealthy (>100) | 1.713 | (1.486–1.975) | <0.001 | | | | | | | | | | | | |
| **$PM_{2.5}$ (μg/m$^3$, per IQR)** | | | | 1.279 | (1.209–1.353) | <0.001 | 1.297 | (1.224–1.373) | <0.001 | | | | | | |
| **$PM_{10}$ (μg/m$^3$, per IQR)** | | | | | | | | | | 1.279 | (1.217–1.344) | <0.001 | 1.289 | (1.226–1.356) | <0.001 |
| **$NO_X$ (ppb, per IQR)** | | | | 0.743 | (0.696–0.794) | <0.001 | | | | 0.756 | (0.710–0.805) | <0.001 | | | |
| **$NO_2$ (ppb, per IQR)** | | | | | | | 0.718 | (0.665–0.774) | <0.001 | | | | 0.735 | (0.685–0.790) | <0.001 |
| **$O_3$ (ppb, per IQR)** | | | | 0.838 | (0.774–0.906) | <0.001 | 0.874 | (0.812–0.942) | <0.001 | 0.856 | (0.793–0.924) | <0.001 | 0.895 | (0.832–0.962) | 0.003 |
| **Temperature (℃, per IQR)** | 1.238 | (1.146–1.338) | <0.001 | 1.165 | (1.077–1.262) | <0.001 | 1.147 | (1.060–1.241) | 0.001 | 1.182 | (1.092–1.280) | <0.001 | 1.165 | (1.076–1.261) | <0.001 |
| **Relative humidity (%, per IQR)** | 1.195 | (1.117–1.278) | <0.001 | 1.136 | (1.054–1.224) | 0.001 | 1.147 | (1.066–1.235) | <0.001 | 1.184 | (1.099–1.277) | <0.001 | 1.196 | (1.111–1.287) | <0.001 |
| **Sex** *(Reference: Female)* | | | | | | | | | | | | | | | |
| Male | 1.102 | (1.010–1.202) | 0.028 | 1.108 | (1.013–1.211) | 0.025 | 1.113 | (1.019–1.216) | 0.018 | 1.118 | (1.022–1.222) | 0.014 | 1.124 | (1.029–1.227) | 0.010 |
| **Age group (y)** *(Reference: <24)* | | | | | | | | | | | | | | | |
| 25–44 | 1.023 | (0.920–1.139) | 0.673 | 1.046 | (0.937–1.168) | 0.420 | 1.038 | (0.930–1.157) | 0.508 | 1.048 | (0.940–1.170) | 0.397 | 1.041 | (0.934–1.160) | 0.469 |
| 45–64 | 1.398 | (1.247–1.567) | <0.001 | 1.382 | (1.228–1.555) | <0.001 | 1.392 | (1.239–1.564) | <0.001 | 1.401 | (1.246–1.575) | <0.001 | 1.409 | (1.255–1.582) | <0.001 |
| >64 | 1.767 | (1.517–2.058) | <0.001 | 1.757 | (1.502–2.055) | <0.001 | 1.752 | (1.500–2.047) | <0.001 | 1.757 | (1.503–1.054) | <0.001 | 1.748 | (1.497–2.042) | <0.001 |
| **Time of crash** *(Reference: Day)* | | | | | | | | | | | | | | | |
| Evening (18:00–23:59) | 0.975 | (0.882–1.078) | 0.623 | 1.033 | (0.932–1.144) | 0.538 | 1.082 | (0.976–1.199) | 0.135 | 0.988 | (0.892–1.095) | 0.818 | 1.030 | (0.930–1.141) | 0.571 |
| Night (00:00–05:59) | 1.491 | (1.254–1.774) | <0.001 | 1.400 | (1.166–1.680) | <0.001 | 1.430 | (1.194–1.711) | <0.001 | 1.398 | (1.168–1.675) | <0.001 | 1.428 | (1.195–1.705) | <0.001 |
| **Scene-to-hospital arrival time (min)** | 0.999 | (0.998–0.999) | <0.001 | 0.998 | (0.998–0.999) | <0.001 | 0.998 | (0.998–0.999) | <0.001 | 0.998 | (0.998–0.999) | <0.001 | 0.998 | (0.998–0.999) | <0.001 |
| **Type of road user** *(Reference: Motorcyclist)* | | | | | | | | | | | | | | | |
| Bicyclist | 1.457 | (1.183–1.795) | <0.001 | 1.429 | (1.154–1.770) | 0.001 | 1.409 | (1.139–1.744) | 0.002 | 1.400 | (1.130–1.733) | 0.002 | 1.381 | (1.116–1.708) | 0.003 |
| Pedestrian | 1.379 | (1.180–1.612) | <0.001 | 1.412 | (1.202–1.660) | <0.001 | 1.406 | (1.198–1.649) | <0.001 | 1.440 | (1.227–1.688) | <0.001 | 1.437 | (1.228–1.683) | <0.001 |
| Auto occupant | 0.185 | (0.133–0.256) | <0.001 | 0.180 | (0.128–0.253) | <0.001 | 0.182 | (0.130–0.255) | <0.001 | 0.190 | (0.137–0.263) | <0.001 | 0.192 | (0.139–0.265) | <0.001 |

| | | | | | | | | | | | | | | | |
|---|---|---|---|---|---|---|---|---|---|---|---|---|---|---|---|
| **Road width (m)** | 1.014 | (1.009–1.019) | <0.001 | 1.013 | (1.008–1.019) | <0.001 | 1.014 | (1.008–1.019) | <0.001 | 1.014 | (1.008–1.019) | <0.001 | 1.014 | (1.008–1.019) | <0.001 |
| Model fit statistics | | | | | | | | | | | | | | | |
| Akaike information criterion | | 13,518.711 | | | 12,763.535 | | | 12,992.112 | | | 12,916.062 | | | 13,148.301 | |
| −2 Log likelihood | | 13,486.711 | | | 12,729.535 | | | 12,958.112 | | | 12,882.062 | | | 13,114.301 | |

The effect estimate was based on the interquartile range (IQR) increases in temperature, relative humidity, and air pollutant concentration. The IQRs of temperature, relative humidity, $PM_{2.5}$, $PM_{10}$, $NO_x$, $NO_2$, and $O_3$ were 9.7 °C, 15%, 15 μg/m$^3$, 26 μg/m$^3$, 19 ppb, 14.6 ppb, and 25 ppb, respectively. AQI, air quality index; CI, confidence interval; IQR, interquartile range; $NO_2$, nitrogen dioxide; $NO_x$, nitrogen oxide; $O_3$, ozone; OR, odds ratio; $PM_{2.5}$, particulate matter ≤ 2.5 μm in diameter; $PM_{10}$, particulate matter ≤ 10 μm in diameter; ppb, parts per billion.

**Table S3.** Results of sensitivity analysis.

| Variables | Model 1 (AQI) | | | Model 2 ($PM_{2.5}$, $NO_X$, $O_3$) | | | Model 3 ($PM_{2.5}$, $NO_2$, $O_3$) | | | Model 4 ($PM_{10}$, $NO_X$, $O_3$) | | | Model 5 ($PM_{10}$, $NO_2$, $O_3$) | | |
|---|---|---|---|---|---|---|---|---|---|---|---|---|---|---|---|
| | OR | 95% CI | *p* | OR | 95% CI | *p* | OR | 95% CI | *p* | OR | 95% CI | *p* | OR | 95% CI | *p* |
| **AQI level** *(Reference: Good (0–50))* | | | | | | | | | | | | | | | |
| Moderate (51–100) | 1.142 | (1.040–1.254) | 0.006 | | | | | | | | | | | | |
| Unhealthy (>100) | 1.750 | (1.516–2.020) | <0.001 | | | | | | | | | | | | |
| **$PM_{2.5}$ (μg/m$^3$, per IQR)** | | | | 1.284 | (1.213–1.360) | <0.001 | 1.300 | (1.226–1.377) | <0.001 | | | | | | |
| **$PM_{10}$ (μg/m$^3$, per IQR)** | | | | | | | | | | 1.289 | (1.225–1.355) | <0.001 | 1.298 | (1.233–1.367) | <0.001 |
| **$NO_X$ (ppb, per IQR)** | | | | 0.734 | (0.685–0.787) | <0.001 | | | | 0.747 | (0.698–0.798) | <0.001 | | | |
| **$NO_2$ (ppb, per IQR)** | | | | | | | 0.721 | (0.668–0.778) | <0.001 | | | | 0.737 | (0.686–0.792) | <0.001 |
| **$O_3$ (ppb, per IQR)** | | | | 0.840 | (0.776–0.909) | <0.001 | 0.878 | (0.815–0.947) | 0.001 | 0.858 | (0.794–0.926) | <0.001 | 0.898 | (0.835–0.966) | 0.004 |
| **Temperature (°C, per IQR)** | 1.244 | (1.152–1.344) | <0.001 | 1.172 | (1.082–1.268) | <0.001 | 1.155 | (1.067–1.249) | 0.001 | 1.189 | (1.098–1.287) | <0.001 | 1.173 | (1.084–1.269) | <0.001 |
| **Relative humidity (%, per IQR)** | 1.195 | (1.117–1.279) | <0.001 | 1.136 | (1.053–1.225) | 0.001 | 1.148 | (1.066–1.236) | <0.001 | 1.186 | (1.099–1.279) | <0.001 | 1.198 | (1.112–1.291) | <0.001 |
| **Sex** *(Reference: Female)* | | | | | | | | | | | | | | | |
| Male | 1.092 | (1.000–1.192) | 0.050 | 1.097 | (1.003–1.201) | 0.044 | 1.102 | (1.008–1.205) | 0.033 | 1.107 | (1.012–1.211) | 0.027 | 1.112 | (1.017–1.216) | 0.019 |
| **Age group (y)** *(Reference: <24)* | | | | | | | | | | | | | | | |
| 25–44 | 1.020 | (0.916–1.136) | 0.673 | 1.042 | (0.933–1.164) | 0.466 | 1.033 | (0.926–1.154) | 0.559 | 1.045 | (0.936–1.167) | 0.432 | 1.038 | (0.930–1.158) | 0.507 |
| 45–64 | 1.409 | (1.256–1.581) | <0.001 | 1.395 | (1.239–1.571) | <0.001 | 1.405 | (1.250–1.581) | <0.001 | 1.412 | (1.255–1.589) | <0.001 | 1.420 | (1.264–1.596) | <0.001 |
| >64 | 1.773 | (1.519–2.068) | <0.001 | 1.767 | (1.508–2.070) | <0.001 | 1.754 | (1.499–2.052) | <0.001 | 1.761 | (1.504–2.062) | <0.001 | 1.743 | (1.491–2.039) | <0.001 |
| **Time of crash** *(Reference: Day)* | | | | | | | | | | | | | | | |
| Evening (18:00–23:59) | 0.970 | (0.877–1.073) | 0.551 | 1.028 | (0.927–1.140) | 0.538 | 1.077 | (0.970–1.194) | 0.164 | 0.983 | (0.887–1.090) | 0.818 | 1.026 | (0.925–1.138) | 0.507 |
| Night (00:00–05:59) | 1.492 | (1.250–1.781) | <0.001 | 1.417 | (1.177–1.707) | <0.001 | 1.449 | (1.207–1.741) | <0.001 | 1.403 | (1.167–1.687) | <0.001 | 1.434 | (1.196–1.720) | <0.001 |
| **Scene-to-hospital arrival time (min)** | 0.999 | (0.998–0.999) | <0.001 | 0.998 | (0.998–0.999) | <0.001 | 0.998 | (0.998–0.999) | <0.001 | 0.998 | (0.998–0.999) | <0.001 | 0.998 | (0.998–0.999) | <0.001 |
| **Type of road user** *(Reference: Motorcyclist)* | | | | | | | | | | | | | | | |
| Bicyclist | 1.453 | (1.180–1.789) | <0.001 | 1.424 | (1.150–1.765) | 0.001 | 1.406 | (1.136–1.740) | 0.002 | 1.396 | (1.127–1.729) | 0.002 | 1.379 | (1.115–1.706) | 0.003 |
| Pedestrian | 1.376 | (1.177–1.608) | <0.001 | 1.407 | (1.197–1.653) | <0.001 | 1.401 | (1.195–1.644) | <0.001 | 1.436 | (1.224–1.685) | <0.001 | 1.436 | (1.226–1.681) | <0.001 |
| **Road width (m)** | 1.014 | (1.008–1.019) | <0.001 | 1.013 | (1.008–1.019) | <0.001 | 1.013 | (1.008–1.019) | <0.001 | 1.013 | (1.008–1.019) | <0.001 | 1.013 | (1.008–1.019) | <0.001 |

| | | | | | |
|---|---|---|---|---|---|
| Model fit statistics | | | | | |
| Akaike information criterion | 13,196.517 | 12,467.512 | 12,689.858 | 12,600.386 | 12,825.985 |
| −2 Log likelihood | 13,166.517 | 12,435.512 | 12,657.858 | 12,568.386 | 12,793.985 |

The effect estimate was based on the interquartile range (IQR) increases in temperature, relative humidity, and air pollutant concentration. The IQRs of temperature, relative humidity, $PM_{2.5}$, $PM_{10}$, $NO_x$, $NO_2$, and $O_3$ were 9.6 °C, 15%, 15 μg/$m^3$, 26 μg/$m^3$, 20 ppb, 14.6 ppb, and 25 ppb, respectively. AQI, air quality index; CI, confidence interval; IQR, interquartile range; $NO_2$, nitrogen dioxide; $NO_x$, nitrogen oxide; $O_3$, ozone; OR, odds ratio; $PM_{2.5}$, particulate matter ≤ 2.5 μm in diameter; $PM_{10}$, particulate matter ≤ 10 μm in diameter; ppb, parts per billion.
